# Type I Interferons, Autophagy and Host Metabolism in Leprosy

**DOI:** 10.3389/fimmu.2018.00806

**Published:** 2018-04-23

**Authors:** Thiago Gomes Toledo Pinto, Leonardo Ribeiro Batista-Silva, Rychelle Clayde Affonso Medeiros, Flávio Alves Lara, Milton Ozório Moraes

**Affiliations:** ^1^Leprosy Laboratory, Oswaldo Cruz Institute, Rio de Janeiro, Brazil; ^2^Laboratory of Cellular Microbiology, Oswaldo Cruz Institute, Rio de Janeiro, Brazil

**Keywords:** leprosy, tuberculosis, innate immunity, autophagy, type I interferon, metabolism, host-directed therapy

## Abstract

For those with leprosy, the extent of host infection by *Mycobacterium leprae* and the progression of the disease depend on the ability of mycobacteria to shape a safe environment for its replication during early interaction with host cells. Thus, variations in key genes such as those in pattern recognition receptors (*NOD2* and *TLR1*), autophagic flux (*PARK2, LRRK2*, and *RIPK2*), effector immune cytokines (*TNF* and *IL12*), and environmental factors, such as nutrition, have been described as critical determinants for infection and disease progression. While parkin-mediated autophagy is observed as being essential for mycobacterial clearance, leprosy patients present a prominent activation of the type I IFN pathway and its downstream genes, including *OASL, CCL2*, and *IL10*. Activation of this host response is related to a permissive phenotype through the suppression of IFN-γ response and negative regulation of autophagy. Finally, modulation of host metabolism was observed during mycobacterial infection. Both changes in lipid and glucose homeostasis contribute to the persistence of mycobacteria in the host. *M. leprae*-infected cells have an increased glucose uptake, nicotinamide adenine dinucleotide phosphate generation by pentose phosphate pathways, and downregulation of mitochondrial activity. In this review, we discussed new pathways involved in the early mycobacteria–host interaction that regulate innate immune pathways or metabolism and could be new targets to host therapy strategies.

## Introduction

Leprosy is caused by *Mycobacterium leprae* or *Mycobacterium lepromatosis*. Here, we will discuss mechanisms of infection and host–pathogen interaction mediated by *M. leprae*. The heaviest exposed population, including the household and family members and social contacts of patients, is considered to have the highest risk of developing leprosy, but the disease will not necessarily progress during their lifetime. Thus, mycobacterial infection is a necessary, but not sufficient cause of leprosy progression. During the natural course of the disease, it has been suggested that once *M. leprae* infects an individual through the airways, the bacteria can come into the lungs and be phagocytosed by resident macrophages. The mycobacteria can infect epithelial cells in the nasal mucosa and penetrate the organism, while host cells initiate an innate response to eliminate the pathogen (1). Intracellular mycobacteria are able to use different strategies to circumvent potential bactericidal peptides: (i) mimic a viral response; (ii) upregulate lipid metabolism; or (iii) downregulate pro-inflammatory cytokines, which is generally associated with a cascade of pro-mycobacteria events (2–4). These virulence strategies are related to other pathogens, such as *Mycobacterium tuberculosis*, suggesting that virulent mycobacteria can share common mechanisms of host colonization (5–7). Thus, by understanding the critical pathways related to the subversion of antimicrobial responses, researchers can understand the conditions for successful mycobacterial infection and, perhaps, the disease progression. Actually, these novel pathways, which use different strategies during *M. leprae* infection have already been described, but a better understanding of these phenomena could help us interfere, reverse or halt the disease progression.

In this regard, the *M. leprae* genome is highly conserved, and the strain circulating worldwide has remained basically the same for the past 1,000 years (8). So, the decline of leprosy in Europe does not account for genetic changes in *M. leprae* that could impact bacterial virulence. Currently, it is clear that very few differences are observed between strains isolated from different clinical forms of the disease. One possible conclusion is that the various stages and clinical forms observed in cases of leprosy are similar due to the host genetics (9).

Large-scale studies have contributed to the identification of new candidate genes and pathways to help understand this complex puzzle. These strategies provide insights not only about leprosy but also about other immune-based and/or infectious diseases. In fact, the most successful genome-wide association studies (GWASs; or genomic scans) were performed in leprosy, as compared, for example, to tuberculosis studies, in which no genes were consistently pinpointed. Several genes have been associated with leprosy, such as *NOD2, PARK2/PARCG, LRRK2, RIPK2, TNF/LTA/HLA, LACC1, IL10, TLR*1, and microRNA (miR)-146a (10–14). Single-nucleotide polymorphisms in these genes were replicated consistently in different populations and have been assigned a functional role in leprosy susceptibility. Whole exome sequencing and rare variant analysis have implicated several novel candidates that still need to be validated. Most of these confirmed associations have a modest odds ratio value, but few other infectious diseases have a clear association with key genes that demonstrate consistent results, which can be replicated in populations with different ethnic backgrounds. Interestingly, the most important genes or pathways that emerge after *M. leprae* infections in studies using microarray gene expression are type I interferon (IFN), autophagy and mitochondrial, and lipid metabolism (15). Therefore, different large-scale approaches in the literature are revealing distinct, but complementary pathways that clearly outline the strategies used by *M. leprae* to destabilize antimicrobial responses and establish a safe environment for continuous bacterial replication. We have depicted main pathways associated with disease susceptibility in a way that how could we potentially regulate lipid and mitochondrial metabolism and immuno-inflammatory responses toward a reversion of the phenotype to accelerates treatment and develop new prevention strategies? Hence, in this article, we will discuss seminal findings that reveal critical mechanisms of innate immunity and host metabolism with a direct impact on the disease outcome where modulation could be path toward disease control.

## TLR-2/1-Mediated Antimicrobial Response in Leprosy

In the early stages of mycobacterial infection, macrophages and other cells of the innate immune system are able to rapidly recognize pathogen-associated molecular patterns through exposure to an extensive repertoire of pattern recognition receptors (PRRs). These transmembrane receptors mediate the activation of several signaling pathways in response to intracellular pathogens and initiate important immune events, such as cell differentiation and antimicrobial programs (16). The most recognized toll-like receptors (TLRs) have been observed to mediate the immune recognition of mycobacteria (17). Among these, the TLR-2/1 heterodimer was responsible for recognizing mycobacterial lipoproteins, activating a pro-inflammatory response and releasing vitamin-D-dependent antimicrobial peptides (18). Genetic analysis has demonstrated that polymorphisms in the *TLR1* gene are associated with leprosy susceptibility, and these variations have a functional effect that includes structural modifications to the protein and alterations to TNF/IL-10 log ratio values in the supernatants of *M. leprae*-stimulated peripheral blood mononuclear cells (13, 16). These individual variations exemplify the ability of the host’s immune system to initiate an efficient antimicrobial response against mycobacteria.

Other components also contribute to TLR-2/1 signaling. miR-21, which is highly expressed in the disseminated form of leprosy, it is a suppressive mechanism of host antimicrobial TLR-2/1-mediated genes that affect the production of critical cytokines, such as IL-1β and IL-10 (19). Recently, a novel component of a TLR-2/1-mediated antimicrobial programme has been described. The *S100A12* gene, which encodes the calgranulin C protein, is highly expressed in response to the activation of the TLR-1 receptor. This gene codifies an antimicrobial peptide that is able to kill *M. leprae* directly (20). Also, *S100A12* is more expressed in skin lesions of tuberculoid (TT) patients than in those of lepromatous (LL) patients (20). Since TLR-2/1 signaling pathways regulate this gene, differences in disease susceptibility could be linked to variations of *TLR1* expression and the activation of this signaling pathway among patients and healthy volunteers. Thus, pattern recognition is essential for controlling mycobacterial growth by regulating optimum levels of the TNF/IL-10 ratio during the period of infection, while miR-21 levels could counterbalance or impair an adequate antimicrobial response (19).

## NOD2 Signaling Pathway

In the past few years, independent GWASs in leprosy and inflammatory diseases such as Crohn’s disease (CD) have revealed a common genetic fingerprint and a considerable overlap of susceptibility mechanisms among these pathologies (10, 21, 22). As demonstrated in mycobacterial diseases, the risk variants of inflammatory bowel disease (IBD) comprise genes that are active in the early stages of the host response suggesting that the continuous interaction between host and pathogens shapes genetic factors that are predisposed to IBD (23). An important signaling pathway identified by a GWAS was the nucleotide-binding oligomerization domain containing 2 (NOD2)-mediated immune response, where variants of genes involved in this signaling pathway are also implicated in susceptibility to *M. leprae* infection and CD (10).

NOD2 is an intracellular component of NOD-like receptors that detects muramyl dipeptide (MDP), which is a cell wall structure. *M. leprae* presents a distinct MDP compared with other mycobacteria (24). However, even with these structural modifications, *M. leprae* MDP maintains the capacity to trigger the NOD2 response. Upon recognizing MDP, NOD2 is able to initiate a leucine-rich repeat kinase 2 (LRRK2)-dependent pro-inflammatory response, as well as other cellular processes, such as autophagy (25). LRRK2 is a downstream component of NOD2 signaling, which enhances the inflammatory cytokine production that is required for antimicrobial activity in the presence of macrophages (25). For this reason, the *LRRK2* gene is similar to the many critical genes involved in the NOD2-mediated response that is associated with leprosy susceptibility, CD, and Parkinson’s disease (PD) (10). Unbalanced LRRK2 activity is related to excessive inflammation, which leads to tissue damage. It has been reported that a specific mutation in the *LRRK2* gene is associated with acute inflammation in both leprosy and CD cases, supporting the assumption that these diseases share common pathological mechanisms (26). Furthermore, a recent study has found that functional variations in *LRRK2* genetically link CD to PD, affecting cellular processes such as kinase activity and autophagy (27).

**Figure 1:**
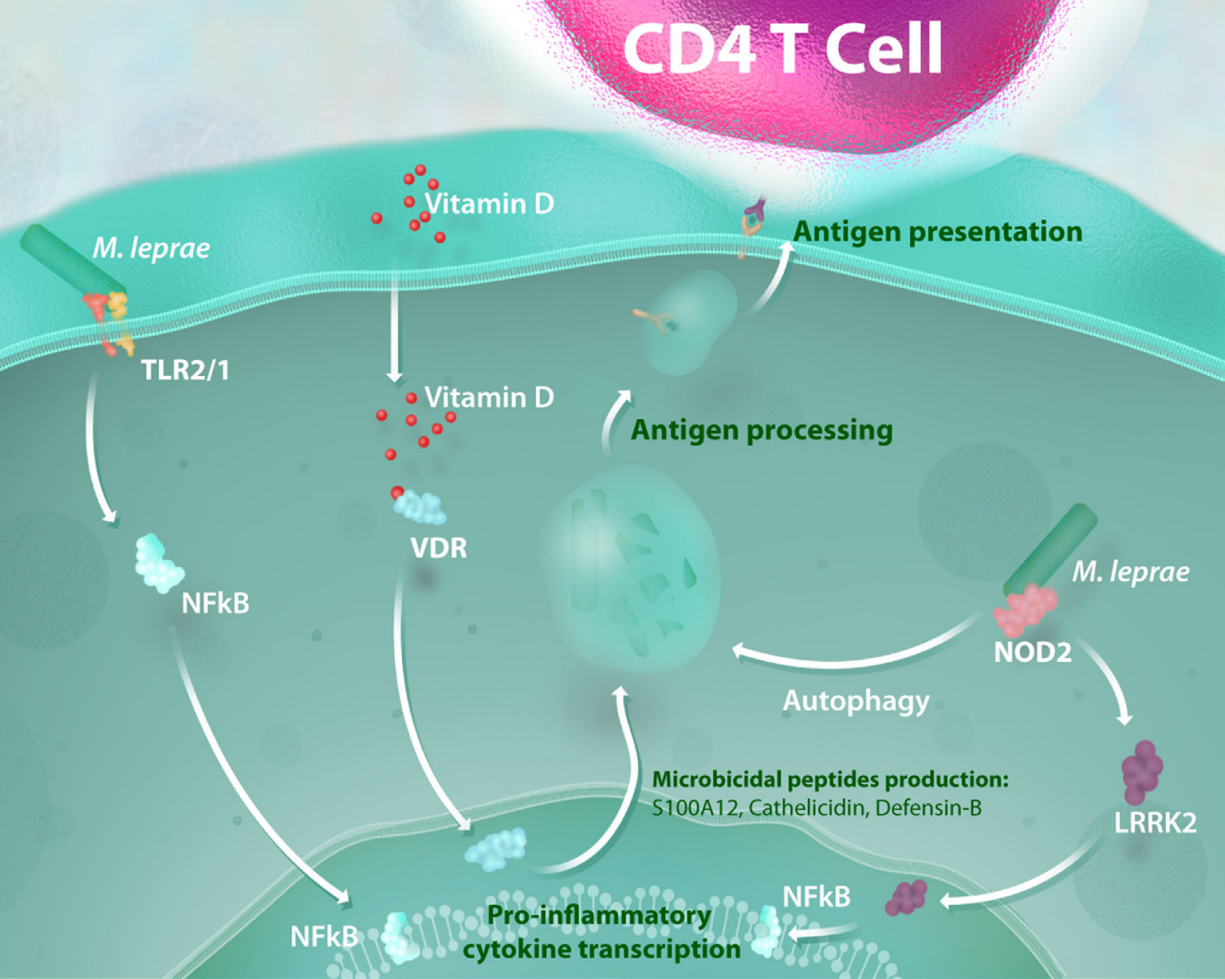
Antimicrobial activity and NOD2-induced autophagy mediate the link between innate and adaptive immunity in mycobacterial infection. The recognition of mycobacterial lipoproteins by the TLR-2/1 heterodimer is a critical way to initiate a pro-inflammatory response and activation of a vitamin-D antimicrobial program against intracellular pathogens such as *Mycobacterium leprae*. Mycobacterial muramyl dipeptide sensing by NOD2 receptors enhances the inflammatory response in a leucine-rich repeat kinase 2 (LRRK2)-dependent manner and activates autophagic mechanisms. All of these processes lead to mycobacterial killing and are essential for bacterial handling, antigen presentation, and consequent generation of an effective CD4 T cell response.

In like manner, genetic variation at *NOD2* is reported to be associated with exacerbated inflammatory responses in leprosy reactions that could modulate downstream pathways, such as LRRK2 activation (28). Notably, NOD2 activation induces the differentiation of monocytes into dendritic cells (DCs) in an IL-32-dependent manner (29). This DC activation triggers autophagy, a process required for bacterial handling, antigen presentation and generation of CD4 T cell response (30). Individuals suffering from CD present a defective activation of these processes, which are still poorly investigated in leprosy. In addition to the genetic relevance of the NOD2 response to leprosy susceptibility, some advances in functional studies have demonstrated that this signaling pathway is upregulated in patients with paucibacillary leprosy when compared with those that manifest the disseminated (multibacillary) form of the disease (29). These findings show that the activation of the NOD2 response is an essential link between innate and adaptive immunity, and aberrant NOD2 signaling results in impairment of antimicrobial activity and defective antigen presentation in leprosy (Figure 1).

## Type I IFN and Autophagy: The Heterogeneity of DNA Sensing in Mycobacterial Infections

In parallel, other mycobacterial components trigger innate immune responses. A classical view of phagocyte–mycobacteria interaction supports the view that virulent bacilli are able to persist within phagosomes, preventing their fusion with lysosomes to achieve a safe environment for replication (31, 32). This interpretation has been extended and updated in light of new data, which suggest that a breach in the phagosomal membrane and cytosol contamination during the course of an infection leads to a permissive response (6). Mechanisms of phagosome maturation are arrested and punctured during mycobacterial infection, which involves virulence factors that manipulate important host response against intracellular infection.

The ESX-1 secretion system is a determinant of mycobacterial virulence that is presented in pathogenic mycobacteria, such as *M. tuberculosis* and *M. leprae*, and it is responsible for the secretion of (CFP-10) and early secreted antigenic target 6 kDa (ESAT-6) proteins (5). The absence of this secretion system in virulent mycobacteria such as *Mycobacterium bovis* BCG supports the importance of those proteins for the success of mycobacterial infection (33). Just after infection, virulent mycobacteria express the ESX-1 system, exporting ESAT-6, which is able to create a fissure in the phagosomal membrane (34). Consequently, ESX-1-mediated pore formation allows an equalization of phagosomal and cytosol content. This process is essential for bacteria to acquire nutrients from the host cell and deliver virulence factors capable of downregulating host responses against the pathogen (5, 35). The leakage of mycobacterial DNA from phagosomes into the cytosol strongly activates the host cell cytosolic surveillance pathways, triggering both a type I IFN response (6) and autophagy (36, 37), which comprise pro- and antibacterial responses, respectively (6). Furthermore, ESX-1 activity and cytosolic recognition of mycobacterial DNA is also involved in the activation of caspase-1, promoting the formation of the inflammasome complex and regulation of IL-1β secretion (38, 39).

Type I IFN (IFN-α/β) activation was originally characterized as a pathway involved in controlling virus infection. However, in the past decade, a number of reports have described a type I IFN transcriptional signature in the pathogenesis and progression of tuberculosis (40) among other mycobacterial diseases. The production of IFN-β may inhibit IL-1β activation, which plays a critical role in the elimination of *M. tuberculosis* (41). IFN-β-mediated suppression of the host bactericidal mechanisms is also noticed in leprosy. An inverse correlation between IFN responses (type I and II) is observed in the clinical spectrum of leprosy. Paucibacillary patients preferentially express type II IFN (IFN-γ) and, consequently, its downstream antimicrobial genes, preventing the spread of mycobacteria; by contrast, the IFN-β program is prominent in multibacillary patients (42). The IFN-β response can induce IL-27-dependent IL-10 activation, which in leprosy, is a well-known immune suppressive mechanism that favors mycobacterial growth and dissemination (43).

Interferon-β induction is redundant, and it involves a large repertoire of nucleic acid sensors (44). *M. tuberculosis* models have been used to generate most of the existing data on type I IFN trigger mechanisms for infections, and this area has not been fully explored in leprosy studies. Once released into the cytosol, extracellular mycobacterial DNA ligates to a double-strand DNA sensor (6). In this context, different studies reported that cyclic GMP-AMP synthase (cGAS) is the primary sensor for mycobacterial DNA (39, 45, 46). After DNA recognition, cGAS is able to produce the second messenger cyclic GMP-AMP, a potent ligand of the stimulator of interferon genes (STING), TANK-binding kinase 1 (TBK1), interferon regulatory factor 3 (IRF3) signaling pathway exhibiting a transcriptional profile of the type I IFN response that antagonizes the host’s antimicrobial programmes (6).

Conversely, cGAS-mediated DNA sensing and STING/TBK1 activation is also required for mycobacterial targeting of the ubiquitin-dependent autophagy pathway, an efficient mechanism that eliminates intracellular pathogens and links innate and adaptive immune responses by enhancing antigen presentation (37, 45, 47). However, only one-third of the intracellular mycobacteria in the host are delivered for autophagic degradation, suggesting that virulent mycobacteria have an active mechanism to evade autophagy (36). The paradoxical mechanisms of DNA sensing during mycobacterial infection are not clearly understood, but they involve a type of bifurcation that could be dependent on multiplicity of infection. Thus, it is likely when infected by a low number of mycobacteria, the host can preferentially load autophagy and control the infection. If a higher mycobacterial burden is presented during infection, a pro-mycobacteria response is directed.

Many factors may be involved in the heterogeneity of DNA sensing following infection. Determining the immunological status of a host at the early stages of host–pathogen is critical to define the course of infection. An initially permissive environment favors bacterial colonization and triggers virulent mechanisms. The increase of the mycobacterial burden and consequent virulence released into the host cell contribute to an imbalance of the DNA-mediated response, driving type I IFN production that, in turn, leads to an impairment of the host antimicrobial mechanisms (2, 6). Host genetic variation in the PRRs of genes that mediate mycobacterial interactions could also modulate the bacilli uptake (9), as well as the activation of an inflammatory response that directly affects downstream signaling pathways, such as cGAS/STING signaling. However, in large-scale screenings, no evidence has been found that major genes or consistent effects in this pathway are associated with leprosy. Mutations in *TMEM173*, which encode STING, are related to selective STING activity, and such activity is able to disrupt IRF3 phosphorylation without affecting other activities of TBK1 (48). These findings support the hypothesis that variation in genes that encodes key DNA sensing components may contribute to the heterogeneity of DNA-mediated responses. Previous research suggests that other cytosolic sensors, such as AIM2 inflammasome, may interact competitively with the mycobacterial DNA implicated in the balance of STING-mediated responses (49).

**Figure 2:**
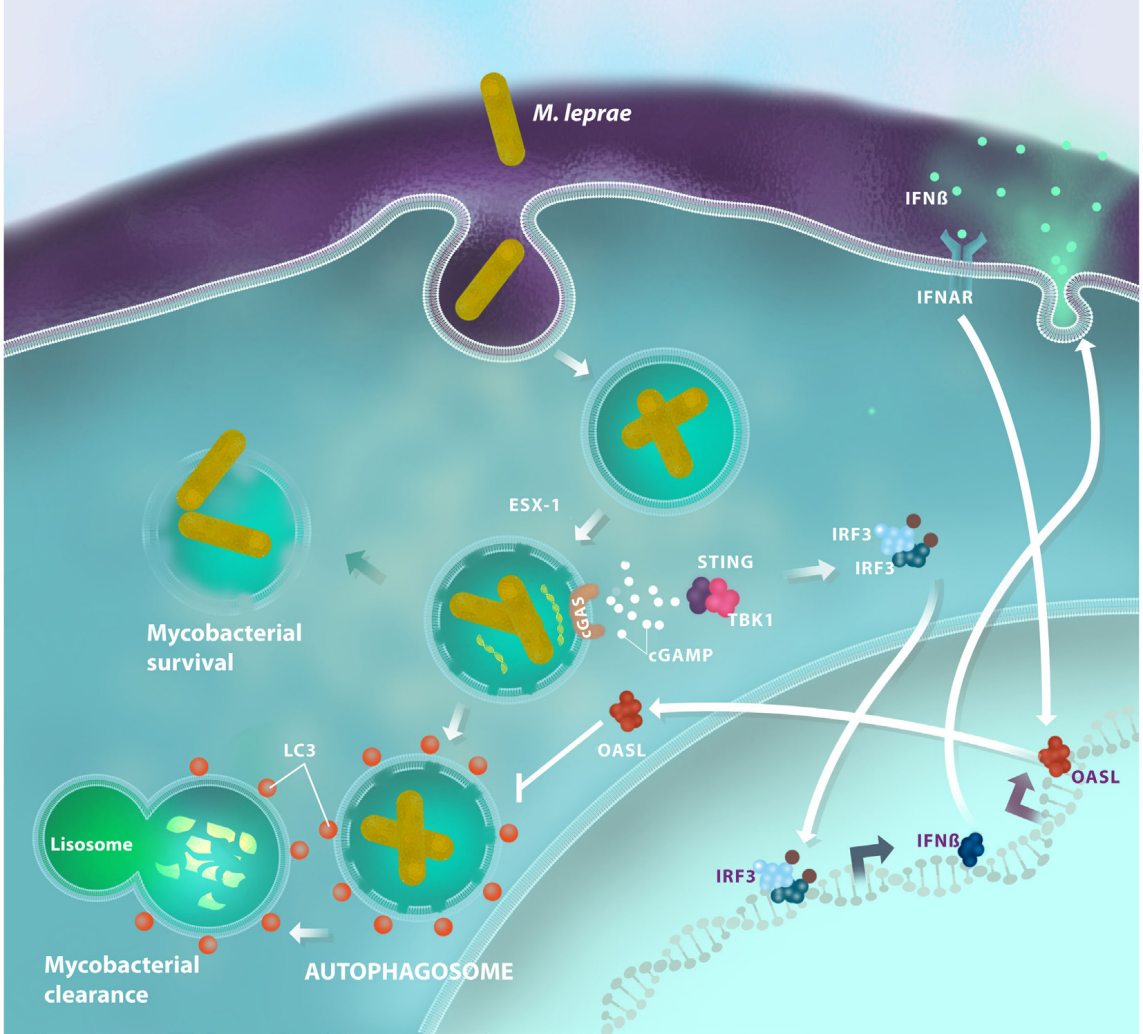
Antimicrobial autophagy is inhibited in *Mycobacterium leprae* infections through the activation of the type I interferon (IFN) pathway. After being inside the host cell, *M. leprae* is able to disrupt the phagosomal membrane by a mechanism that is dependent on the mycobacterial ESX-1 secretion system. Then, bacterial DNA activates the cyclic GMP-AMP synthase (cGAS)/stimulator of interferon genes (STING)/TANK-binding kinase 1 (TBK1) pathway and promotes interferon regulatory factor 3 (IRF3) translocation, which induces IFN-β production. In response to an autocrine and/or paracrine IFN-β stimulus, macrophages increase *OASL* expression. OASL production inhibits bacterial clearance, blocking LC3-dependent autophagy, and promotes mycobacterial survival by creating a permissive microenvironment for sustainable growth and disease progression.

The targeting and delivery of *M. tuberculosis* for autophagic degradation occurs by a recruitment of the host’s ubiquitin chains, a process that depends on Parkin (*PARK2*), an E3-ubiquitin ligase (37). Intracellular *M. tuberculosis* avoid ubiquitin or proteasomal host systems. More than one decade ago, the gene *PARK2*, which encodes Parkin, was associated with leprosy susceptibility (11); this suggests that Parkin also controls ubiquitination and autophagy levels during *M. leprae* infection. A more recent study has showed that multibacillary patients demonstrated autophagy impairment, while paucibacillary ones exhibited strong autophagy upregulation (50). The research revealed how live *M. leprae* actively downregulates the autophagic machinery of human monocytes to generate a protected intracellular niche for bacterial replication. Following this research, our group described a transcription profile of the type I IFN response in both human Schwann cells and macrophages following *in vitro* infection with live *M. leprae*. *OASL* [2′-5′-oligoadenylate synthetase (OAS) like] was the most differentially expressed interferon-stimulated gene in our study (2). OASL is a member of the OAS family, a group of proteins with a recognized antiviral action, although its function in bacterial infections is poorly understood. OASL can play a dual role following activation: the ubiquitin-like domain of OASL can interact with RIG-I, a double-strand RNA sensor, leading to type I IFN activation enhancement (51). Conversely, viral double-stranded DNA can induce an OASL-mediated type I IFN inhibitory effect by blocking cGAS/STING signaling (52). Upon *M. leprae* infection, macrophages are able to produce high levels of OASL in a STING-dependent manner. This production is associated with the persistence of *M. leprae* inside the cell as OASL inhibits autophagic mechanisms that are essential for mycobacterial clearance (2) (Figure 2). However, the mechanisms for the OASL-mediated blockage of autophagy need to be explained. The OASL–cGAS interaction, as it occurs during double-stranded DNA virus infection, could also be investigated in mycobacterial infection to improve our understanding of how OASL modulates cGAS/STING-mediated autophagy. Moreover, investigating the interactions of OASL with other molecules in its ubiquitin-like domain may help us understand the role of OASL in the regulation of immune responses against intracellular infections. Thus, these data suggest that OASL participates in the fine-tuning of infection outcomes by regulating DNA sensing pathways.

## Metabolic Immunity in Leprosy

Using microarray analysis, researchers have pointed out important changes in metabolic pathways in bacterial infections such as *M. leprae* (15, 53). Determining the ability of intracellular pathogens to modulate the host metabolic pathways has provided an understanding of the infection in pathogenic terms (54). *M. leprae* must adjust the cytosol to its requirements, and the breach of the phagosomal membrane releases bacterial components that will trigger a metabolic switch.

When infected by intracellular pathogens, immune cells are able to increase nitric oxide synthetase inducible (iNOS) and indoleamine 2,3-dioxygenase-1 (IDO-1) activity. These enzymes catalyze the degradation of l-arginine and l-tryptophan, respectively, resulting in local amino acid deprivation (55). While iNOS generates nitric oxide radicals, IDO-1 leads to the production of kynurenine metabolites (56). This metabolite activates the aryl hydrocarbon receptor, promoting the conversion of naive CD4 T cells into Foxp3+ regulatory T cells (57). DCs are able to increase *IDO1* expression and activity in response to IFN-γ (56), and *IDO1* is highly activated in leprosy patients (58). Genetic variations in the *IDO1* gene are related to differential activation of regulatory T cell function and correlated with autoimmune disease development (59). IDO-1-mediated l-tryptophan deprivation is an innate response against viral replication. However, it is ineffective against mycobacterial infection. Despite the drastic reductive evolution in the *M. leprae* genome, all enzymes involved in l-tryptophan anabolism have been maintained. *M. leprae* infection activates the IDO-1 signaling pathway (55, 60, 61) in an iron and IL-10-dependent manner. Thus, the l-tryptophan deprivation does not affect *M. leprae* survival (56). Transforming growth factor beta, which is highly expressed in leprosy patients (58), is able to maintain high IDO-1 expression in DCs through phosphorylation of its immune-based inhibitory tyrosine motifs, leading to a sustained immunoregulatory effect (62).

**Figure 3:**
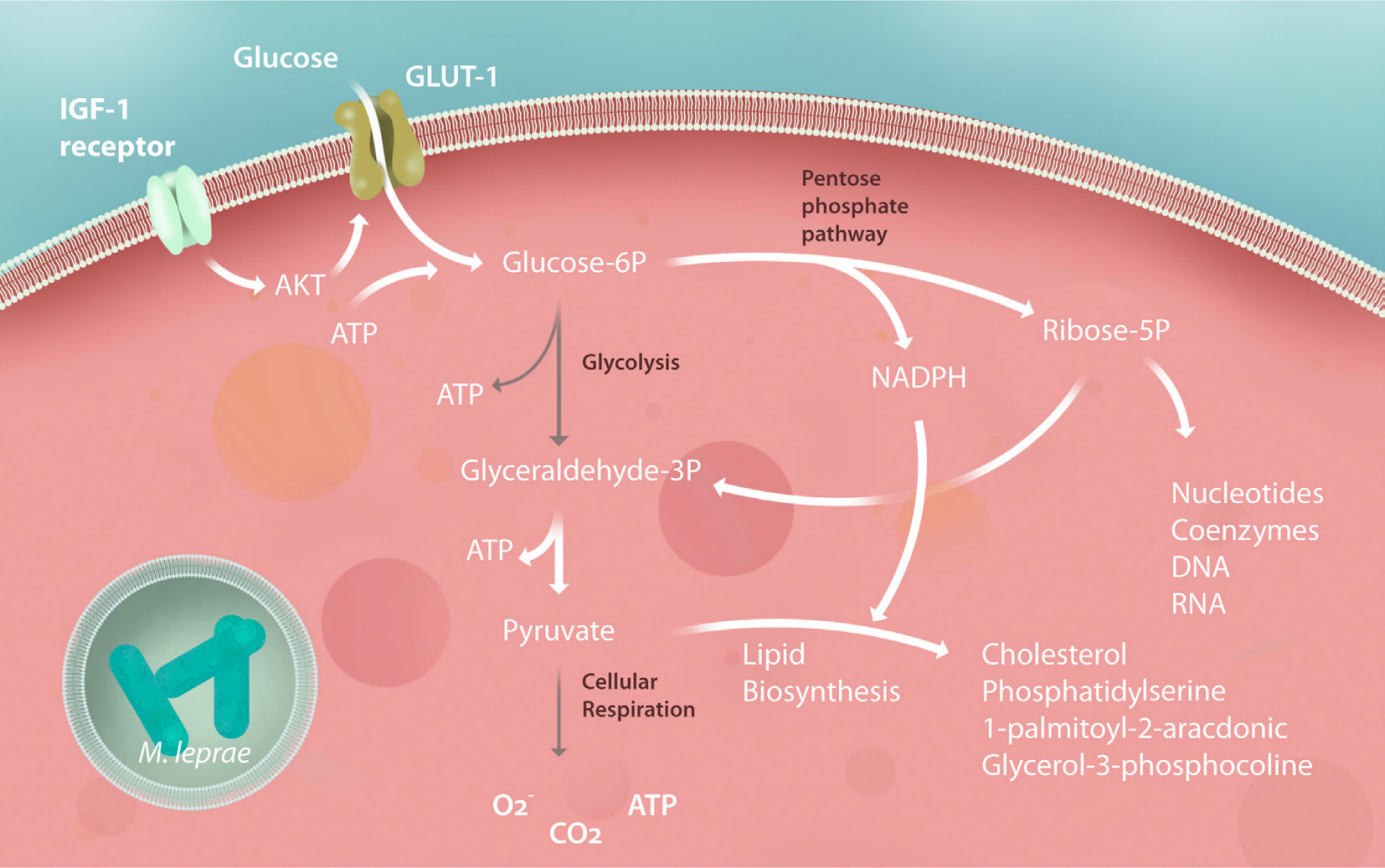
Schwann cell central metabolism is subverted by *Mycobacterium leprae*. After infection, Schwann cells increase their insulin-like growth factor (IGF) expression, upregulating glucose transporter 1 (GLUT-1) and glucose uptake by Akt signaling. Glycolysis is downregulated, feeding the pentose phosphate pathway (PPP) with carbons used to synthesize building blocks to promote Schwann cell dedifferentiation and proliferation, generating during this process the reducing power [nicotinamide adenine dinucleotide phosphate (NADPH)] responsible for pumping up lipid biosynthesis. Pyruvate generated by the PPP is rapidly converted to citrate and subsequently converted to lipids, virtually stopping the tricarboxylic acid cycle, respiration and mitochondrial energy potential of the Schwann cells. All of these modulations are crucial for subverting the host immunity against the mycobacteria and, consequently, to the success of the *M. leprae* infection, representing potential for new host-target therapy strategies to halt leprosy progression. The gray arrows represent downregulated pathways.

Glucose plays a central role in energy metabolism as a carbon source. In addition, glucose is a highly versatile precursor of amino acids, coenzymes, fatty acids, and cholesterol. After phosphorylation, this molecule can follow a catabolic pathway such as that of glycolysis, generating energy and carbon to be burned in the mitochondria. Alternately, it can follow an anabolic pathway, such as the pentose phosphate pathway (PPP), which generates carbons and reducing equivalents, in the form of nicotinamide adenine dinucleotide phosphate (NADPH) to synthesize lipids, nucleotides, and aromatic amino acids (63). In both leprosy and tuberculosis, it was found that the bacilli increases glucose uptake in the infected host cells in a glucose transporter 1-dependent manner (64, 65). Modulation of glucose metabolism was noticed in *M. leprae*-infected Schwann cells (64) (Figure 3) while this event has been demonstrated in human macrophages infected by *M. tuberculosis* (65). The hypothesis that these mechanisms also occur in *M. leprae-*infected macrophages needs to be investigated.

Studies on the host carbohydrate metabolism during infection have demonstrated that many pathogens, including viruses such as immunodeficiency virus (HIV), hepatitis C virus (HCV), Mayaro, transmissible gastroenteritis virus, and human cytomegalovirus, can increase host cell glucose uptake to provide biosynthetic precursors for their replication (66–71). Furthermore, the synthesis of immune-active lipids, such as 1-palmitoyl-2-arachidonoyl-sn-glycero-3-phosphorylcholine, is able to generate a strong anti-inflammatory response when oxidized (72). In *M. leprae* infection, live bacteria are able to avoid free radical generation by using carbons from the electron transport chain (ETC) for lipid synthesis (64). To support the positive feedback of this pathway, *M. leprae* mediates an increase in the production of insulin-like growth factor (IGF)-1 in both macrophages and Schwann cells (73). IGF-1 is one of the main regulators of glycolysis metabolism. In macrophages, IGF-1 can impair the host antimicrobial activity and increase lipid metabolism (73). Otherwise, IGF-1 shares a high amino acid homology with insulin (74), and the structure of its receptors is closely related to post-receptor signaling (75). This signaling activation involves glucose uptake with subsequent lipid synthesis and storage in lipid bodies. Indeed, glucose uptake can be positively modulated by the IGF-1 receptor through the activation of the PI3K signaling pathway. Thus, virulent mycobacteria cause a metabolic switch that drives the cell toward the production of several micronutrients, macronutrients, and electron acceptors in response to infection.

After *M. leprae* infection, Schwann cells redirect glucose from the glycolysis pathway to the PPP through the activation of G6PD, increasing the carbon flux to lipid synthesis. The PPP generates ribose-5-phosphate and NADPH, as the main products that sustain cell proliferation, lipid biosynthesis, and the regeneration of oxidized glutathione, which is the main free radical scavenger of human cells (63). *M. leprae* is highly dependent on the host PPP because G6PD inhibition by pharmacological interference and RNA interference associated with G6PD knockdown decreases the viability of intracellular mycobacteria (64). During its adaptation, *M. leprae* has developed another mechanism to live inside human cells: shutting down the cell’s mitochondria (64). The dissipation of the mitochondrial inner membrane electric potential after infection demonstrates the suppression of the ETC. This is probably due to the redirection of carbons to lipid synthesis for the formation of lipid bodies in infected cells, and it will increase long chain fatty acids in cytosol, responsible to mitochondrial permeability transition pore opening and consequent electric potential dissipation (3, 64, 76).

Gene expression analysis of skin lesions of lepromatous patients revealed upregulation of *SREBF1*, a host gene involved in lipid synthesis (77). Together with this observation, a mass spectroscopy analysis revealed that these patients’ skin lesions were enriched with cholesterol (77) and other immune-active lipids, such as oxidized 1-palmitoyl-2-arachidonoyl-sn-glycero-3-phosphorylcholine (oxPAPC), prostaglandins E2 and D2, lipoxin A_4_, and omega-3 and omega-6 (72, 78). Live *M. leprae* can actively induce and support adipophilin, adipose differentiation related protein, and perilipin expression in macrophages, promoting lipid accumulation within the phagosome (77). In this context, host lipid synthesis and its deposition in infected tissues have been associated with pathogenesis and infection success in leprosy (79), with special involvement of cholesterol. In contrast to *M. tuberculosis, M. leprae* is not able to use cholesterol as a carbon source (80). However, during the reductive evolution of the genome, *M. leprae* maintained an enzyme of paramount importance to its survival, 3β-hydroxysteroid dehydrogenase, which is a catalyst in the first step of cholesterol degradation: the oxidation of cholesterol to cholest-4-en-3-one (cholestenone) (80). In clinical applications, avoiding cholesterol synthesis by treating infected macrophages with statins, inhibitors of HMG-CoA reductase, has a strong impact on intracellular *M. leprae* and *M. tuberculosis* viability (81). Based on microscopy data from our previous study, in which we demonstrated the ability of *M. leprae* to recruit and surround itself with lipid bodies (3), we hypothesized that *M. leprae* could use lipids to cover and hide its surface antigens from innate immune receptors in the cytosol.

Altogether, these host metabolic alterations are essential for immune response modulation and infection success. For that reason, new strategies based on host metabolite identification could, in the near future, contribute to preclinical diagnosis. The development of fast, highly sensitive, and non-invasive diagnostic tests is paramount for the control of this disease. As an example, it was demonstrated that it is possible to identify leprosy patients through detection of leukotriene E4 by gently pressing silica plates against their skin for a few seconds (82). Based on the fact that *M. leprae*-infected Schwann cells increase their glucose uptake by about 40% (64), we propose, as another example, that full body imaging of the glucose analog fludeoxyglucose using positron emission tomography could represent a potential non-invasive alternative for diagnosing pure neural leprosy.

## Lipid Metabolism Deregulation Associated with Inflammation in Leprosy

Several diseases are associated with deregulation of the host lipid metabolism, favoring an exacerbated inflammatory process that contributes to immunopathogenesis. In an experimental model of arteriosclerosis, for example, the lipid accumulation process and atherosclerotic plaque development are mediated by the production of monocyte chemoattractant protein-1 (MCP-1), which recruits monocytes to the inflammatory site. Largely differentiated from anti-inflammatory macrophages with an M2 profile, which has a foamy phenotype, these monocytes are rich in lipid droplets (83). MCP-1-mediated recruitment of peripheral monocytes was also observed in a zebra fish model of *Mycobacterium marinum* infection. In this model, MCP-1 produced by infected resident macrophages actively participated in the recruitment of monocytes to the infection site by a mechanism that was dependent on the STING signaling pathway (84). In the context of *M. leprae* infection, in the absence of *OASL*, a gene induced by type I IFN, there is a drastic decrease in the levels of MCP-1 and the intracellular viability of the bacilli in *M. leprae*-infected macrophages (2). Indeed, MCP-1 induction can be mediated by STING either by a type I IFN-dependent pathway or by an independent pathway (85–87). These data, taken together, suggest a scenario in which the induction of the type I IFN pathway participates in MCP-1 induction. The enhancement of MCP-1 aids the recruitment of monocytes at the site of infection and promotes the differentiation of monocytes into macrophages with a M2 phenotype, exhibiting high levels of IL-10 and prostaglandin E2 (PGE2) (88). Lipid bodies are sites of production of eicosanoids, such as PGE2, leukotriene B4 (LTB4), and lipids, including cholesterol. This could explain the characteristic phenotype of foamy macrophages that present in the skin lesions of patients with lepromatous leprosy, as well as the abundance of immunological mediators, such as IL-10, IL-4, PGE2, and MCP-1, in these lesions (89).

In a *M. tuberculosis* murine model, IL-1β triggered PGE2 production as a protective response toward mycobacterial clearance and it is also negatively regulated type I IFNs. Curiously, highly susceptible mice (IL-1β knockouts, for example) can be rescued using PGE2 and zileuton, which is an inhibitor of 5-lipoxygenase that blocks LTB4 and, consequently, TNF (41, 90). Genetic polymorphisms of *LTB4* demonstrate an important association with the development of severe tuberculous meningitis, in which the inadequate balance of the inflammatory response that is mediated by TNF and LTB4 may aggravate the disease progression (90). The importance of the host’s lipid metabolism regulation, which can affect the availability of nutrients to the pathogen as well as the production of inflammatory mediators, is increasingly evident. Host-based therapies are currently under development with the goal of metabolic drugs that could be interesting adjuvants in the mycobacterial diseases treatment, such as leprosy and tuberculosis.

Thus, ongoing mycobacterial survival is associated with enhancements to lipid metabolism. After infection takes place, mycobacteria cause a shift in the host cell gene expression that leads to lipid uptake through the receptor induction of cholesterol (15, 77) and the formation of lipid bodies (91). Strong modulation of lipid synthesis pathways in host cells by *M. leprae* or *M. tuberculosis* has been observed, and it has been suggested that lipid droplets work as a nutrient reservoir for *M. tuberculosis* (7). Although *M. leprae* are unable to remove carbons from cholesterol (80), both *M. leprae* and *M. tuberculosis* seem to take shelter within lipid bodies, which are formed abundantly by host cells (91). Therefore, as an example, a pharmacological approach to compensate for the induction of this crucial pathway for *M. leprae* survival would be the use of statins as an adjuvant in combination with multidrug therapy. Results from experimental models (81) suggest that modulation of autophagic mechanisms could also promote the antimicrobial response against *M. tuberculosis* and decrease inflammation-mediated immunopathology (31, 82, 83). Recently, mammalian target of rapamycin pharmacological agents, including rapamycin or AMPK targets such as metformin, have been tested in clinical trials as an adjuvant therapy in tuberculosis; these tests have been successful and can be applied in leprosy (92–94).

## Conclusion

An infectious disease is a result of a specific and successive combination of events that can only culminate in complete progression if the bacteria are able to block several restrictive antimicrobial mechanisms. The last 10 years of research have been remarkable for revealing novel genes associated with leprosy, including complementary approaches such as genomic scans or GWASs and microarray analysis. Combining these data produce a clear overview of the mechanisms induced by bacteria to survive within hostile and sterile cellular cytosol. Gene-sensing mycobacterial components such as *NOD2* and *TLR1* and pathways that regulate autophagy (*PARK2, LRRK2*, and *RIPK2*) are intrinsically antimicrobial, but they can be opposed and inhibited by the emergence of type I IFN induction. In this scenario, double-stranded DNA receptors and STING/TBK1/IRF3 signaling drive a pro-mycobacterial response.

The fact remains that it is very difficult to define the chronology of these events or even the precise moment when the disease progression takes place in the infected individual. The rationale here is that defining these steps carefully and observing the fine-tuning of genotypic influences on phenotypes can help to halt the disease progression in infected people. Consequently, the current challenge is to combine results from *in vitro* and genotype-to-phenotype studies toward the development of host-directed therapies.

## Author Contributions

TP, LB-S, FL, and MM contributed equally to this manuscript. RM contributed with the “Metabolic immunity in leprosy” topic. All authors participated in the conception, design and writing of this review.

## Conflict of Interest Statement

The authors declare that the research was conducted in the absence of any commercial or financial relationships that could be construed as a potential conflict of interest.
